# Challenges in homology search: HMMER3 and convergent evolution of coiled-coil regions

**DOI:** 10.1093/nar/gkt263

**Published:** 2013-04-17

**Authors:** Jaina Mistry, Robert D. Finn, Sean R. Eddy, Alex Bateman, Marco Punta

**Affiliations:** ^1^EMBL-European Bioinformatics Institute, Wellcome Trust Genome Campus, Hinxton, Cambridge, CB10 1SD, UK, ^2^Wellcome Trust Sanger Institute, Wellcome Trust Genome Campus, Hinxton, Cambridge, CB10 1SA, UK and ^3^HHMI Janelia Farm Research Campus, 19700 Helix Drive, Ashburn, VA 20147, USA

## Abstract

Detection of protein homology via sequence similarity has important applications in biology, from protein structure and function prediction to reconstruction of phylogenies. Although current methods for aligning protein sequences are powerful, challenges remain, including problems with homologous overextension of alignments and with regions under convergent evolution. Here, we test the ability of the profile hidden Markov model method HMMER3 to correctly assign homologous sequences to >13 000 manually curated families from the Pfam database. We identify problem families using protein regions that match two or more Pfam families not currently annotated as related in Pfam. We find that HMMER3 E-value estimates seem to be less accurate for families that feature periodic patterns of compositional bias, such as the ones typically observed in coiled-coils. These results support the continued use of manually curated inclusion thresholds in the Pfam database, especially on the subset of families that have been identified as problematic in experiments such as these. They also highlight the need for developing new methods that can correct for this particular type of compositional bias.

## INTRODUCTION

### Known problems in detecting homology via sequence similarity

Homology, or evolutionary descent from a common ancestor, is widely used as a basis to transfer structural and functional annotation between proteins (1–3) and to study protein evolution (4). Homology is most often inferred by establishing statistical significance of observed similarity in protein sequence alignments. Several methods based on sequence–sequence, sequence–profile or profile–profile alignments have been developed to identify evolutionary-related protein sequences (5–10). Current challenges in homology detection via sequence similarity include problems with homologous overextension of alignments (11) and regions of amino acid compositional bias under convergent evolution (12). Note that the expression ‘compositional bias’ is often used to indicate a bias in amino acid frequencies within a protein sequence region, that is, a deviation from the frequencies typically observed in globular soluble proteins. An example of this bias is the highly polar, highly charged intrinsically disordered regions (13). Convergent evolution, however, may also lead to a different type of bias, which is reflected not in amino acid frequencies but rather in the way amino acids are distributed along the sequence. The heptad repeats that characterize canonical coiled-coils represent a typical example of this bias (14).

### Benchmarking HMMER3 E-values estimates using Pfam

Here, we test the ability of the profile hidden Markov model (profile-HMM) method HMMER3 [http://hmmer.janelia.org/) to correctly infer homology. To this end, we use a library of >13 000 profile-HMMs taken from the Pfam database of protein families (15). Pfam’s comprehensiveness (the fact that Pfam models annotate a large proportion of all residues in UniProt Knowledgebase, UniProtKB (16)] allows us in many cases to identify sources of discrepancies in HMMER3 E-value estimation without using a benchmark. For example, we can look at cases where Pfam annotation, at a given significance threshold, would classify the same region of a sequence into two or more families thought to be evolutionarily unrelated. We call this ‘overlap analysis’. Benchmarks are typically derived from protein structure database classifications, but solved protein structures are depleted for some of the most challenging issues in sequence analysis, such as disordered and/or highly biased sequence composition. Overlap analysis in the entire sequence database reduces this important bias, as Pfam has models representing many such regions, although it has the disadvantage that an overlap does not necessarily imply a false positive (it could be an as-yet unannotated true homology). Nonetheless, manual examination of particular Pfam families that cause an unusual number of overlaps can be used to identify systematic failure modes in HMMER3’s inference of homology.

### Pfam overlaps

We define overlaps as sequence regions that at a given significance threshold simultaneously match two or more Pfam families found in different clans (i.e. families that are not annotated as evolutionary related in the Pfam database). Additionally, we require that ≥50% of the region that matches the profile-HMM of one family also matches the profile-HMM of the other (for at least one of the two families). Overlaps are likely to point to some form of erroneous or incomplete annotation. In particular: (i) the profile-HMMs of one or both of the two families might have been built from a set of non-homologous sequences (incorrect Pfam model); (ii) the overlapping families might be evolutionary related and the relationship not yet annotated in Pfam (incomplete Pfam annotation); and (iii) the profile-HMMs might have incorrectly assigned the sequence to one or both of the overlapping families (false positive). We calculate overlaps between families at E-values that are typically used to suggest homology. Although a number of these overlaps will fall into cases (i) to (ii), we expect this set to be enriched in false positives (iii) with respect to all Pfam matches.

## MATERIALS AND METHODS

### Pfam profile-HMM database

Pfam (15) is a database that uses HMMER3 (http://hmmer.janelia.org/) to group homologous protein regions into families. Pfam 26.0 comprises 13 672 families. Each family is represented by a profile-HMM generated with HMMER3 using a manually curated ‘seed’ alignment. The profile-HMM is searched against UniProtKB (16), (the Pfam protein sequence database of choice), to find additional homologous sequences. Pfam curators define two separate family-specific significance thresholds for including matches into families, one for domains and one for sequences [a domain can match the same sequence multiple times; see (15) for a detailed explanation of what the different thresholds represent]. Each family is annotated with functional and/or structural information where available. In this study, to simplify our overlap analysis that is based on family-independent significance thresholds, we exclude the 316 families where sequence and domain thresholds differ. These families mostly represent repeats in which the domain threshold is set to be lower than the sequence threshold to capture the highest number of occurrences along individual sequences.

### Definition of overlap

As outlined in the ‘Introduction’ section, overlaps are sequence regions that match two or more Pfam families that belong to different clans, with families in different clans not annotated as related in the Pfam database. To exclude cases that could be resolved by small changes in family boundaries, we consider only overlapping regions that cover ≥50% of the alignment co-ordinates of at least one of the two families involved. Note: alignment coordinates represent the region of the sequence for which HMMER3 can produce a reliable alignment in contrast to envelope coordinates that define the region for which there is substantial probability mass to support a homologous match. Thus, the envelope coordinates span a region that is equal or wider than the alignment coordinates.

### Calculating Pfam family overlaps

A subset of Pfam 26.0 profile-HMMs (13 356, see earlier) was searched against UniProtKB release 2011_06 using HMMER3.0 hmmsearch with default parameters. E-values are as reported by HMMER3 hmmsearch, with no additional correction for having performed multiple profile-HMM searches. We define a match as a domain alignment that has an E-value less than or equal to a specified significance threshold. We calculate overlaps between different Pfam families’ matches, as defined earlier in the text, for three different E-value thresholds: 0.001, 0.01 and 0.1.

### Calculating expected number of false positives

The expected number of false positives (FP) can be calculated using the equation:
(1)
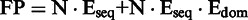

where N is the number of Pfam families, E_seq_ is the per sequence E-value threshold and E_dom_ is the per domain E-value threshold. As we excluded families that in Pfam had different sequence to domain significance thresholds, in this study sequence and domain E-value thresholds were always the same. The total number of Pfam families N that we considered here was 13 356. It follows that, for example, the expected number of false positives at an E-value ≤0.01 was 13 356 × 0.01 + 13 356 × 0.01 ×0.01=135.

### Winner-takes-all greedy algorithm for assigning overlapping domains to families

We compiled a list that contained all overlapping domains, and a list of overlaps these domains were involved in (note: a domain could be involved in overlaps with multiple other domains; therefore, in a family, the number of overlapping domains was lower than or equal to the number of overlaps). We selected the family with the highest number of overlapping domains and assigned these overlapping domains to it (at E-value 0.01, this would be PF04156 IncA having 308 overlapping domains, see ‘Results’ section). We recalculated the list of overlapping domains in each remaining family after removing all overlaps that involved the first family. In particular, domains that overlapped only with domains in the first family would disappear from the list. Families left with no overlapping domains would also be removed. Among the remaining families, we again selected the one with the highest number of overlapping domains and repeated all previous steps. We iterated this procedure until no more overlapping domains (and families) were left. We also experimented with assigning the overlap to the domain with the highest E-value instead of to the family with the highest number of overlapping domains.

### Per family count of overlapping clans

When considering the number of overlapping clans in a family, we counted clan overlaps for each family domain after the greedy algorithm was applied. Where a single domain overlapped with multiple clans, all clans were counted. Note that if a family was not a member of a clan, it was treated as being in a clan with only one member family (i.e. itself).

### Assigning transmembrane, coiled-coil and disordered region labels to Pfam families

The presence of transmembrane helices, coiled-coil and disordered regions was predicted in the seed alignments of all families. Transmembrane helices were predicted using Phobius with default parameters (17), coiled-coil regions were predicted using ncoils with default parameters (http://www.russelllab.org/cgi-bin/coils/coils-svr.pl) and disordered regions using IUPred with the long option (18). For each seed alignment, we determined whether ≥50% of seed member regions had 20 consecutive residues predicted to be coiled-coil, 20 consecutive residues predicted to be disordered and ≥2 predicted transmembrane helices. Families that satisfied these constraints were labelled as coiled-coil, transmembrane (order is important) and disordered. Additionally, we also identified seed alignments that had 50 consecutive predicted coiled-coil regions in ≥50% of seed members. Note that in these calculations, predicted coiled-coil, transmembrane or disordered residues falling outside of the family seed regions were not taken into consideration. Hence, for example, a family covering a soluble domain of an α-helical integral membrane protein would not be labelled as transmembrane.

### Null2 model corrections

The HMMER3 hmmsearch output gives a bias composition correction score for each sequence and domain, which is the bit score difference contributed by the null2 model (see ftp://selab.janelia.org/pub/software/hmmer3/3.0/Userguide.pdf). The sequence and domain scores include this bias composition correction. A high bias score may indicate a false positive, especially if the bias score is larger than the overall bit score. We took the domains that had E-value ≤0.01 and calculated the proportion of families that had >50% of domains with a bias score/bit score ratio >0.1.

## RESULTS

### Few families contain most domains with overlaps

We take 13 356 families from Pfam release 26.0 (15) (see ‘Materials and Methods’ section for the criterion used to exclude some families), and run their corresponding profile-HMMs against UniProtKB (16). We count the number of domains with overlaps generated when using three different E-value thresholds for establishing significance: 0.001, 0.01 and 0.1. A winner-takes-all greedy algorithm is used for assigning domains with overlaps to families so that each such domain belongs to only one family (‘Materials and Methods’ section).

**Figure 1.**
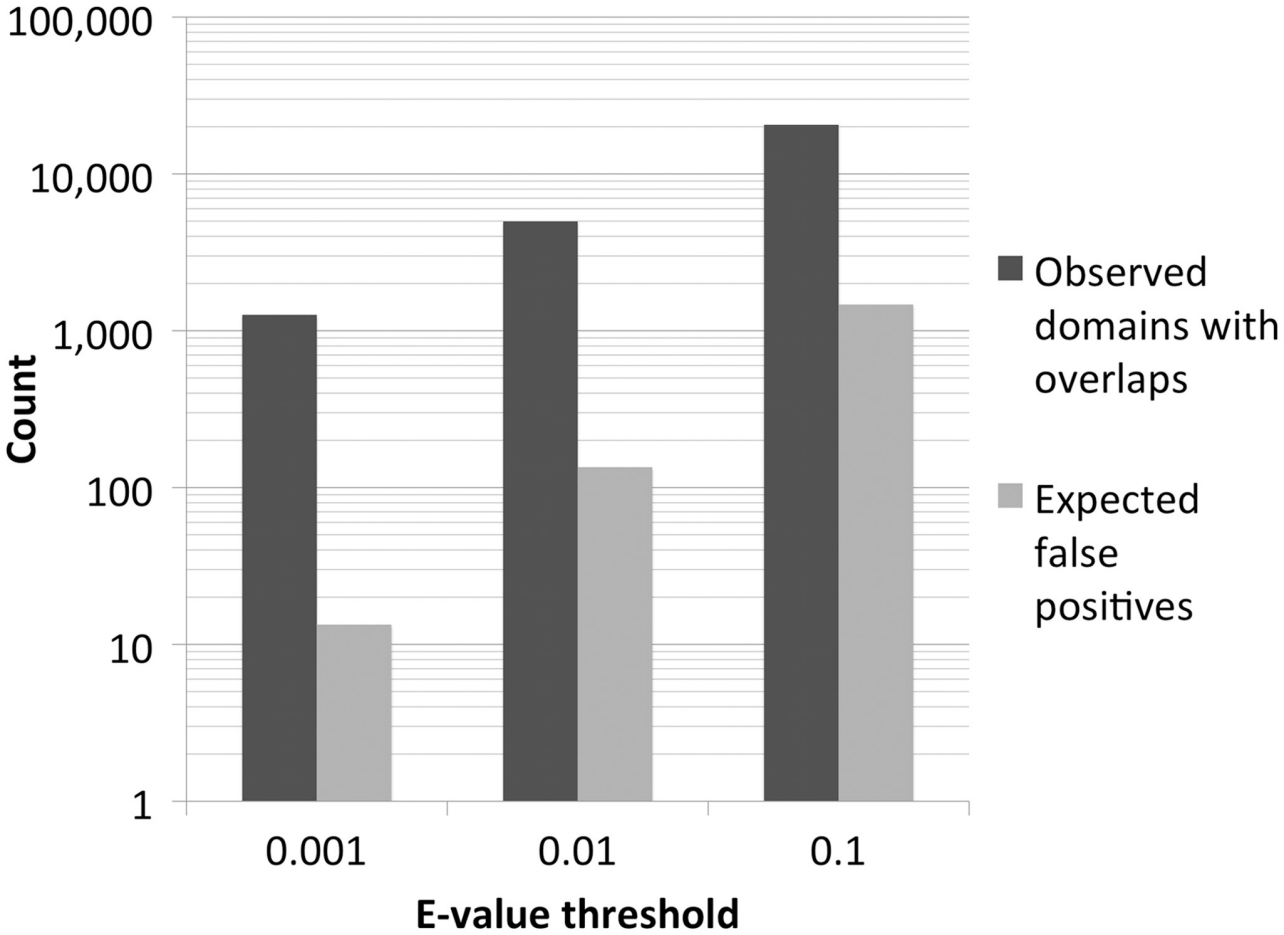
Observed number of overlapping domains (dark grey) and expected number of false positives (light grey) at three different E-value significance thresholds for the 13 356 Pfam families considered here.

In Figure 1, on a logarithmic scale, we report the total number of observed domains with overlaps and the number of expected false positives based on the E-value thresholds used for significance [see Equation (1) in ‘Materials and Methods’ section]. We see that domains with overlaps largely exceed expected false positives at all reported thresholds. Note, however, that because not all such domains will correspond to false positives (‘Introduction’ section), a direct comparison between these numbers cannot be drawn.

**Figure 2.**
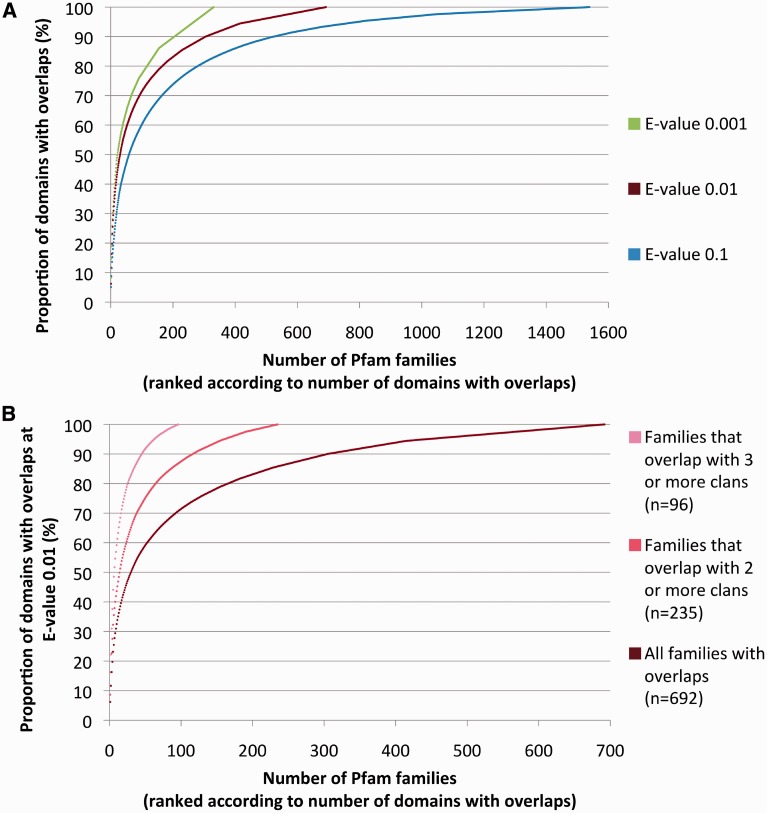
(**A**) Cumulative proportion of overlapping domains in Pfam families. Families are ranked according to the number of their domains that overlap (in descending order) after applying a winner-takes-all greedy algorithm that assigns overlapping domains to families (see ‘Materials and Methods’ section). Data shown for three E-value significance thresholds. (**B**) Same as 2A red line, with additional plots for families that overlap with two or more, and three or more clans only (E-value = 0.01).

Next, we rank families according to the number of member domains with overlaps, from highest to lowest, and plot the cumulative proportion of overlaps (Figure 2A). We see that although overlapping families may be numerous, the majority of member domains with overlaps are found in a small number of families. For E-values of 0.001, 0.01 and 0.1, we find that 50% of domains with overlaps are found in 22, 30 and 59 families (of 13 356), respectively. At the same significance thresholds, 75% of domains with overlaps are found in 87, 124 and 214 families.

### Coiled-coil regions and, in part, transmembrane regions are overrepresented in families overlapping with many clans

We now focus on families that overlap with multiple clans (‘Materials and Methods’ section). As, from a Pfam perspective, these families seem to be particularly promiscuous, we hypothesize that this set is more likely to have a high false-positive enrichment rate (although it may also include some of the most interesting cases of as yet undetected evolutionary relationships). Hereafter, for the sake of simplicity, we report results only for E-value 0.01 (see Supplementary Materials, ‘GreedyByDomain E-value 0.01’ tab, for the complete list of overlapping families). Results for E-value 0.1 and 0.001 follow similar trends (data not shown). In Figure 2B, we plot the cumulative distribution of overlaps for families that overlap with two or more, or three or more clans. For comparison, the cumulative distribution for all overlapping families is also shown (dark red, same as in Figure 2A). In total, 235 families overlap with two or more clans and 96 families overlap with three or more clans.

**Figure 3.**
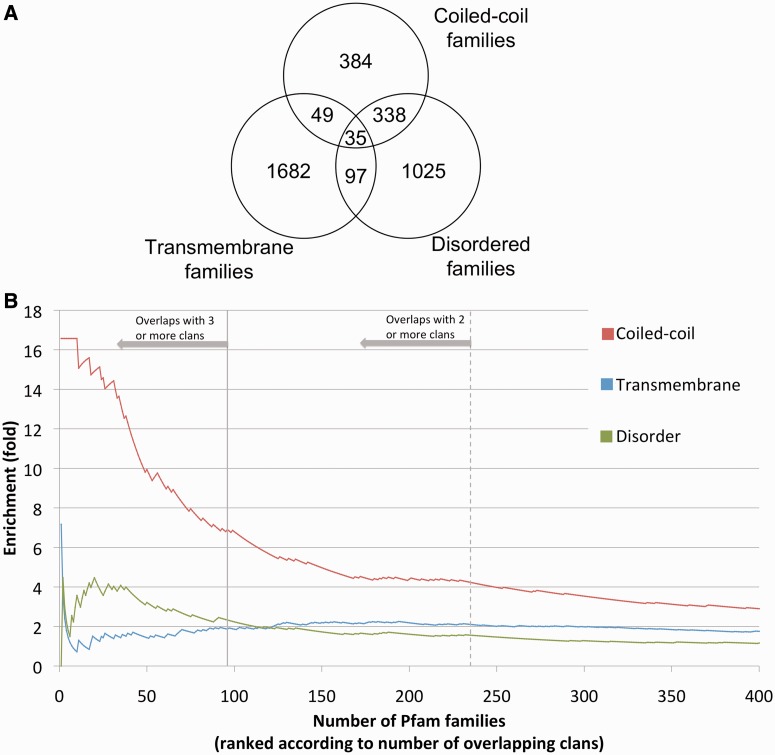
(**A**) Venn diagram with overlap between families predicted to be coiled-coil, disordered and transmembrane (see ‘Materials and Methods’ section) as observed in 13 356 total families. Coiled-coil: consecutive coiled-coil regions of 20 residues predicted in ≥50% of seed member regions. Disordered: consecutive intrinsic disordered regions of 20 residues predicted in ≥50% of seed member regions. Transmembrane helices: ≥2 transmembrane helices predicted in ≥50% of seed members regions. (**B**) Overrepresentation of predicted coiled-coil, transmembrane helices and instrinsic disorder when considering Pfam families with overlapping domains versus all Pfam families. Overlaps are calculated with respect to an E-value significance threshold of 0.01. Families are sorted by the number of clans they overlap with (descending) after a winner-takes-all greedy algorithm for assigning overlapping domains to families is applied. Note: two/three or more means two/three or more clans other than the one the family belongs to. Overrepresentation at each point *x* in the *x* axis is obtained by calculating the proportion of families with a given label (e.g. coiled-coil) among the first *x* families and dividing by the proportion of all families (*n* = 13 356) with that label. Note that for the sake of simplicity, we truncated the *x*-axis at 400 families. Labels assigned to families as described in 3A.

We next assign, to each family, labels that correspond to three predicted sequence features, all of which represent different types of amino acid compositional bias. In brief, if at least 50% of the seed alignment regions of a family are predicted to have two or more transmembrane helices, the family is labelled as transmembrane; if the same percentage of seed sequence regions are predicted to have ≥20 consecutive amino acids in a coiled-coil region or in an intrinsically disordered region, the family is labelled as coiled-coil or disordered, respectively (‘Materials and Methods’ section, see also Supplementary Materials, ‘labels for all families’ tab, for list of labels). The same family may receive multiple labels (Figure 3A). Note that the seed sequence regions are the manually curated set of representative sequences that are aligned and used to build Pfam families’ profile-HMMs. In Figure 3B, we rank all overlapping families according to the number of clans they overlap with (from most to least, left to right) and look at overrepresentation of each of the aforementioned regions with respect to what was observed in all 13 356 families considered here. Coiled-coil families are overrepresented >4-fold in families that overlap with two or more clans (crossing point between the solid red line and the dashed grey vertical line) and almost 7-fold in families that overlap with three or more clans (crossing point between the solid red line and the solid grey vertical line). Overall, we can assign a coiled-coil label to 42% of families that overlap with three or more clans. Overrepresentation correlates with predicted coiled-coil length: 19% of all families that overlap with three or more clans can be labelled as coiled-coil based on predicted consecutive regions of ≥50 amino acids (instead of ≥20 as used elsewhere in this article), accounting for a 45-fold enrichment with respect to all 13 356 families considered here.

**Figure 4.**
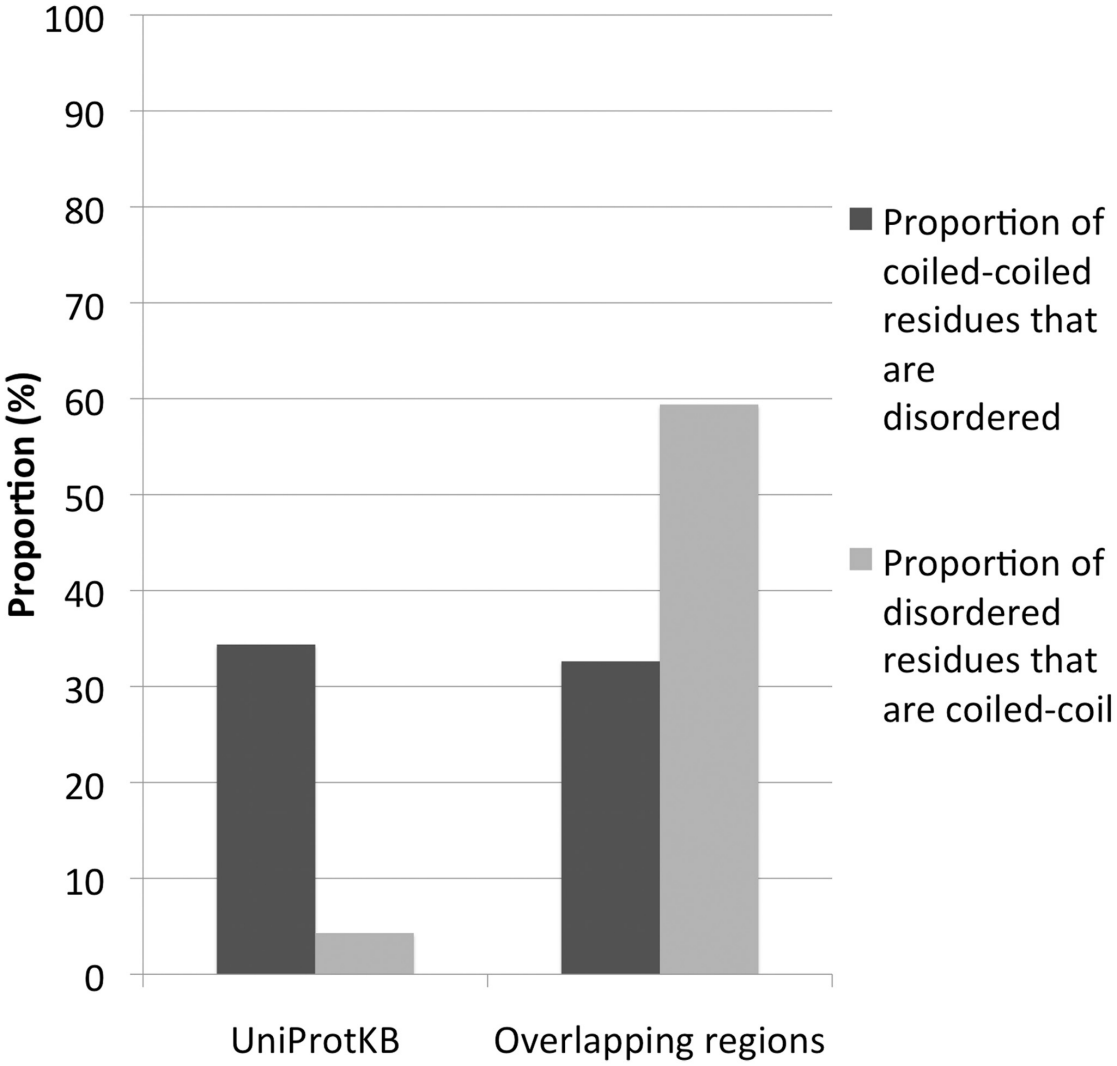
Comparison between the proportion of residues predicted to be in coiled-coil and in disordered regions (dark and light grey, respectively) in overlaps versus the proportion in UniProtKB (version 2011_06).

Predicted transmembrane families are also overrepresented but to a much lesser extent than coiled-coil ones (∼2-fold, Figure 3B). Predicted disordered families are overrepresented but with the following caveat. Predicted disordered residues in overlap regions seem to be highly enriched in residues that are also predicted to be coiled-coil (∼59% of cases in overlaps versus ∼4% observed in the entire UniProtKB, Figure 4). The opposite is not true, with the percentage of predicted coiled-coil residues additionally predicted as disordered being similar in overlap regions and in UniProtKB (33% versus 34%). In fact, if we consider only the disordered regions that have no predicted coiled-coil residues, we see no overrepresentation in overlapping families. These data suggest that even in disordered families, it is coiled-coil regions, rather than disordered ones, that correlate with the presence of overlaps.

### Top 20 overlapping families

In Table 1, we report the top 20 families in terms of number of overlapping clans. These families overlap with at least 10 different clans. All can be labelled with one or more of the compositionally biased regions of Figure 3A. In particular, 18 families are labelled as coiled-coil, 4 as transmembrane and 2 as both. Ten families can additionally be labelled as disordered. In most cases, overlap regions are enriched in compositional bias (second to last column in Table 1).

**Table 1.** The 20 Pfam-A families (Pfam 26.0) with the highest number of overlapping clans at an E-value threshold of 0.01, after a winner-takes-all greedy algorithm for assigning overlapping domains to families was applied (see ‘Materials and Methods’ section)

Pfam-A ID (accession)	Description	Number of domains in the family	Number of domains with overlaps	Number of clans the family overlaps with	TMH	CC	DIS	Proportion of residues in overlap regions predicted to be TMH, CC or DIS (%)	Pfam sequence E-value threshold
IncA (PF04156)	Inclusion membrane protein A	1231	308	61	✓	✓	✗	62.6	1.5e-10
Myosin_tail_1 (PF01576)	This family consists of the coiled-coil myosin heavy chain tail region	2383	271	49	✗	✓	✓	82.4	3.0e-11
AAA_13 (PF13166)	Many of the proteins in this family are conjugative transfer protein	986	170	45	✗	✓	✗	77.6	3.0e-09
Reo_sigmaC (PF04582)	A homo trimer, this family features an α-helical triple coiled-coil	456	116	41	✗	✓	✗	61.0	9.8e-08
Baculo_PEP_C (PF04513)	Baculovirus polyhedron envelope protein, C-terminus	189	52	35	✗	✓	✗	62.9	9.9e-07
EzrA (PF06160)	This family represents proteins involved in bacterial cell division	669	28	25	✗	✓	✗	72.7	1.8e-05
DUF827 (PF05701)	Domain of unknown function	474	172	23	✗	✓	✓	43.6	3.3e-05
Filament (PF00038)	This family represents the coiled-coil central rod domain of intermediate filament proteins	2315	35	22	✗	✓	✗	76.3	2.4e-07
DUF869 (PF05911)	Domain of unknown function	246	15	20	✗	✓	✓	78.8	9.6e-08
MFS_1 (PF07690)	This family belongs to the major facilitator superfamily of membrane transporters	175 494	30	19	✓	✗	✗	62.1	2.6e-05
Tropomyosin (PF00261)	Tropomyosin is an α-helical protein that forms a coiled-coil structure of two parallel helices	1389	39	19	✗	✓	✓	80.9	6.5e-06
ATG16 (PF08614)	Autophagy proteins 16 have a dimeric coiled-coil structure	298	26	17	✗	✓	✓	74.3	7.6e-06
MCPsignal (PF00015)	This family represents the coiled-coil bacterial cytoplasmic domain of chemotaxis receptors	20 808	50	17	✗	✓	✗	41.2	3.0e-4
ERM (PF00769)	This family represents the coiled-coil domain of ERM proteins	311	11	15	✗	✓	✓	77.7	3.1e-4
Cenp-F_leu_zip (PF10473)	This family represents a microtubule-binding protein consisting of long coiled-coil regions	138	76	14	✗	✓	✗	60.7	3.4e-4
Spc7 (PF08317)	This family represents proteins involved in cell division in Fungi	183	24	14	✗	✓	✓	69.3	9.2e-06
Tropomyosin_1 (PF12718)	Tropomyosin_1, in the same clan as Tropomyosin aforementioned	964	28	14	✗	✓	✓	92.4	2.9e-4
ABC2_membrane_3 (PF12698)	ABC-2 membrane transporter family	17 995	60	10	✓	✗	✗	34.8	6.3e-05
TPR_MLP1_2 (PF07926)	Proteins in this family feature coiled-coil regions	193	12	10	✗	✓	✓	58.5	2.8e-4
DUF3584 (PF12128)	Domain of unknown function	173	14	10	✓	✓	✓	68.3	1.4e-4

For the purposes of counting the number of clans that a family overlaps with, a family that is not in a clan was counted as being in a clan by itself. Note that ‘number of domains in the family’ is the total number of regions aligned to the family profile HMM with E-value ≤0.01, before clan competition. Columns ‘TMH’, ‘CC’ and ‘DIS’ show whether the families have ≥2 transmembrane helices (TMH), or consecutive coiled-coil regions of 20 residues (CC), or consecutive disordered regions of 20 residues (DIS), predicted in ≥50% of seed member regions. E-values in the last column are calculated from the sequence gathering threshold, i.e. the family-specific bit score significance threshold (15), according to the following formula: *E* = *N* × exp[−λ·(*x* − τ)], where *x* is the bit score gathering threshold, λ and τ are parameters derived from the profile-HMM model (λ is the slope parameter, τ is the location parameter) and *N* is the database size (in this case the size of UniProtKB).

PF04156 (IncA) is the top family both in terms of number of domains with overlaps (308) and of overlapping clans (61). The family is restricted to Chlamydiaceae and is functionally associated with the homotypic fusion of the inclusions of these obligate intracellular parasites (19). Sequences in this family are believed to include two N-terminal transmembrane segments and a coiled-coil region (20). Families with the most overlaps to IncA include membrane families PF00664 (ABC_membrane; 49 overlaps) and PF06305 (DUF1049; 33 overlaps), and coiled-coil families PF04582 (Reo_sigmaC; 45 overlaps), PF13166 (AAA_13; 35 overlaps) and PF01576 (Myosin_tail_1; 29 overlaps). Interestingly, PF04156 (IncA) is the family with the longest HMMER3 tested running time (21). Slowness of HMMER3 on PF04156 is related to the compositional bias of its sequence (21). Among the other families, 10 have well known and characterized members featuring coiled-coil regions. These include PF01576 (Myosin_tail_1) (22), PF04582 (Reo_sigmaC) (23), PF00038 (Filament) (24), PF00261 (Tropomyosin) (25), PF12718 (Tropomyosin_1) (25), PF08614 (ATG16) [see coiled-coil structures 3A7P and 3A70 in the Protein Data Bank (26)], PF00015 (MCPsignal) (27), PF00769 (ERM) (28), PF10473 (Cenp-F_leu_zip) (29) and PF07926 (TPR_MLP1_2) (30). Two families, PF07690 (MFS_1) and PF12698 (ABC2_membrane_3), belong to two of the largest helical membrane protein clans in Pfam. PF13166 (AAA_13) is in the P-loop NTPase clan (CL0023), which includes ATPase family PF00004 (AAA). Although the ATPase domain is not a coiled-coil structure, PF13166 members seem to be bacterial condensin-like proteins that contain a central zinc-hook motif and have an AAA domain that is split by a large coiled-coil region. For the remaining families, little is known experimentally that may confirm or reject the computationally derived hypothesis of them harbouring coiled-coil regions: PF06160 (Ezra) and PF08317 (Spc7) are families involved in cell division, in Firmicutes and Fungi, respectively, and PF04513 (Baculo_PEP_C) is a Baculovirus polyhedron envelope protein. Of the three domains of unknown function in the list, one (PF05701, DUF827) has been recently annotated as containing proteins with weak chloroplast movement under blue light (31), and the new annotation is in Pfam 27.0. Overall, the top 20 overlapping families generate ∼31% of all overlapping domains and 59% of those that occur in families that overlap with three or more clans. As we have seen, all of these families feature predicted coiled-coil or transmembrane regions with the predictions supported in several cases by experimental evidence.

Outside of the top 20 list PF00001 (7tm_1), the seven transmembrane receptor family is the one with the largest number of overlapping domains (231 overlapping domains with four different clans). Of 231 overlaps (note: in this case the number of overlaps is equal to the number of overlapping domains), 226 occur with PF07264 (EI24), a family of multi-span integral membrane proteins believed to be involved in growth suppression and apoptosis in eukaryotes (32).

### Overlapping regions enriched in predicted coiled-coil and transmembrane residues

**Figure 5.**
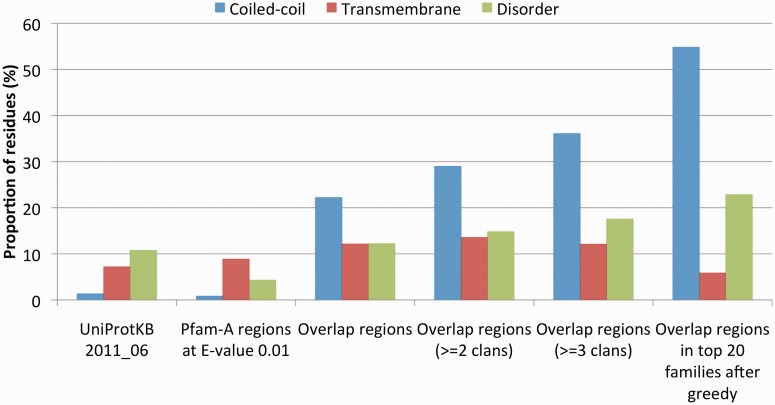
Proportion of residues in predicted transmembrane helices, coiled-coil regions and intrinsically disordered regions in different sets of sequences: UniProtKB (version 2011_06), all domains in the 13 356 Pfam families that we consider in this study, all overlapping regions, overlapping regions of families that overlap with two or more and three or more clans, overlapping regions of the top 20 families in Table 1. Both Pfam domains and overlapping regions are calculated based on an E-value threshold of 0.01.

So far, we have analysed the presence of different types of amino acid compositional bias in the seed regions of families with overlapping domains. This allowed us to see that many families featuring overlapping domains, especially when overlaps occur with multiple Pfam clans, are rich in coiled-coil regions. We now want to verify whether the overlap regions themselves are enriched in residues predicted to be part of coiled-coil or other compositionally biased regions. We see that 22% of residues in all overlap regions are predicted to be coiled-coil (Figure 5, red bars); this is 17 times more than what was observed in UniProtKB and 26 times more than observed in all Pfam domains at an E-value threshold of 0.01. The percentage of residues predicted to be coiled-coil increases for the overlap regions of families that overlap with at least two or three different clans (29 and 36%, respectively). In the top 20 families reported in Table 1, nearly 55% of the residues in the overlapping regions are predicted to be coiled-coil. Overlap regions are also enriched in residues predicted to be in transmembrane helices (Figure 5, blue bars) and in disordered regions (Figure 5, green bars), albeit to a much lower extent. As discussed earlier in the text, overrepresentation of predicted disordered residues seems to be related to the fact that many of these residues are also predicted to be part of coiled-coil regions (Figure 4).

### E-value-based strategy for assigning overlapping domains to families gives similar overrepresentation results

The data we reported in the last two sections are obtained using an E-value-independent protocol to assign overlapping domains to families, when E-values are the quantity that is being tested. We have, however, also experimented with an assignment strategy where the overlapping domain between two families is ascribed to the family that matches with the higher E-value (in other words, the family with the lower E-value is instead awarded the genuine match). This alternative way of assigning overlaps produces overrepresentation curves (data not shown) that show similar trends with respect to the ones in Figure 3B. Predicted coiled-coil regions, for example, are overrepresented 6- and 10-fold in families that overlap with two or more or three or more clans, respectively. In the same way, when compiling the list of top 20 overlapping families, 15 of the 20 families are shared with the list in Table 1. Nineteen families in this alternative top 20 list are labelled as coiled-coil and three are labelled as transmembrane (with two having both labels).

### Monitoring HMMER3 bias correction can help identifying problematic families

One of the two methods that HMMER3 uses to handle sequences that have a biased composition is an unpublished method called null2. The null2 method retests a local sequence comparison under the following null hypothesis: that it was generated from an independent and identically distributed random sequence composition calculated from the weighted mean composition of the profile-HMM’s emission states, within the aligned region. The bias correction, i.e. the bit score difference contributed by the null2 model, is reported as part of the HMMER3 output. In practice, bias corrections that compare in magnitude with final bit score values should be regarded with suspicion and may be indicative of false positive hits. If, for example, we calculate the number of families for which >50% of the member sequences have a bias correction of >10% of their bit score value (member sequences determined according to an E-value threshold of 0.01), we see that 744 of the 13 356 satisfy these constraints. Thirty-six of them are found among the families that overlap with three or more clans (96 families overall), accounting for >6-fold enrichment in this set. Similarly, we observe >5-fold enrichment when considering families such that 75% of sequences have a bias correction >10% of their bit score values. These data indicate that the relative magnitude of the bias correction of a sequence match can provide some indication about the likelihood of matches to be false positives.

## DISCUSSION

Aligning and establishing homology between sequences with amino acid compositional bias is difficult. Sequence comparison methods, including HMMER3, evaluate whether the query and target seem more likely to be homologous (under a model of residue similarity scores), versus random non-homologous sequences. Some low-complexity regions of proteins convergently evolve similar sequences that can score more highly in such sequence comparison than random sequences are expected to. These scores may be ‘statistically significant’, rejecting the null hypothesis (i.e. matches are not recognized as random), but only because the sequences do not fit the simple null hypothesis. This is the reason for so many *ad hoc* methods for low-complexity sequence masking and biased composition score corrections (33–35). HMMER3 has two unpublished biased composition methods. As both are based on an independent and identically distributed random null hypothesis, however, they only model biased sequence composition (i.e. zeroth order effects), not higher order correlations, such as short-period tandem repetitions. Coiled-coil regions, with their characteristic heptad repeat pattern (14), are an example of non-zeroth order correlations in protein amino acid sequences. Patterns of alternating hydrophobic membrane-embedded and polar soluble regions may produce a similar effect in some multi-span membrane proteins. This can explain why families that appear problematic from our analysis often feature coiled-coil or transmembrane regions.

It should be emphasized, however, that our overlap analysis suggests false positive enrichment for only a small fraction of all Pfam families that feature coiled-coil and transmembrane regions. Of 13 356 families considered here, we have classified 806 and 1863 as coiled-coil and transmembrane, respectively. For 89% of coiled-coil and 91% of transmembrane families, we do not observe any overlap when considering an E-value significance threshold of 0.01, leaving us with no evidence of false positives occurring in these families. On the other hand, among the 56 families featuring coiled-coil regions of ≥50 consecutive amino acids, 41% produce overlaps and 32% produce overlaps with three or more clans. This suggests that the longer the coiled-coil region, the more problematic it is for HMMER3 to discriminate between homologues and non-homologues.

Overall, our results suggest that the ‘random sequence’ null hypothesis in the denominator of every method’s log-odds score test [including HMMER3, BLAST (7) and FASTA (5)] is oversimplified. In fact, we expect non-homologous sequences to contain other higher-order features ‘by chance’, such as coiled-coil; hence, perhaps such common convergent features should be included in the null model.

Finally, we note that Pfam relies on manually curated family-specific bit score thresholds for defining significance. One of the leading criteria in defining such thresholds is to avoid overlaps with other, supposedly unrelated families. As a consequence, we can see that thresholds in the top 20 overlapping families (Table 1) tend to be on the conservative side (i.e. correspond to low E-values). This is in line with Pfam goal of minimizing the number of false positives in its families.

## CONCLUSIONS

Several contributing factors may lead sequence alignment programs to mis-identify homologous relationships between proteins. Known problems include homologous overextension (11) and convergent evolution (36) such as the one observed for protein regions with a bias in amino acid frequencies (12). Here, we add to previous studies and use the Pfam collection of manually curated profile-HMMs (15) to test the accuracy with which the alignment program HMMER3 assigns protein sequences to homologous families. We identify a small set of families (representing 1–2% of the total) that, according to our analysis, are likely to be enriched in false positives at E-value significance thresholds generally used to assign homology. We find that sequences in these families often harbour regions that exhibit periodic patterns of compositional bias. These include coiled-coiled and, to a lesser extent, multi-span helical transmembrane domains, both examples of regions that are believed to have arisen independently in non-homologous proteins following convergent evolution. Manual curation of homology thresholds, as implemented in the Pfam database, can help minimize the number of false positives in families with this type of amino acid bias, but development of better bias correction methods is needed to prevent this problem from occurring in automatic database searches.

## SUPPLEMENTARY DATA

Supplementary Data are available at NAR Online.

## FUNDING

Wellcome Trust [WT077044/Z/05/Z]; BBSRC Bioinformatics and Biological Resources Fund [BB/F010435/1]; Howard Hughes Medical Institute (to S.R.E. and R.D.F.); EMBL-European Bioinformatics Institute (to J.M. and A.B.). Funding for open access charge: Wellcome Trust Sanger Institute.

*Conflict of interest statement.* None declared.

## Supplementary Material

Supplementary Data
