# Supplementary material for: Challenges in homology search: HMMER3 and convergent evolution of coiled-coil regions
Source: Nucleic Acids Res. 2013 Apr 17;41(12):e121. doi: 10.1093/nar/gkt263 (PMC3695513; doi:10.1093/nar/gkt263)
Supplement: Supplementary Data [file supp_41_12_e121__index.html]

Challenges in homology search: HMMER3 and convergent evolution of coiled-coil regions — Challenges in homology search: HMMER3 and convergent evolution of coiled-coil regions — Supplementary Data 

# Challenges in homology search: HMMER3 and convergent evolution of coiled-coil regions

## Supplementary Data

files

**Files in this Data Supplement:**

- Supplementary Data - xlsx file
